# Forgotten Joint Score for early outcome assessment after total knee arthroplasty: Is it really useful?

**DOI:** 10.1186/s43019-020-00049-0

**Published:** 2020-07-29

**Authors:** Qunn Jid Lee, Wai Yee Esther Chang, Yiu Chung Wong

**Affiliations:** Total Joint Replacement Center, Yan Chai Hospital, Tsuen Wan, Hong Kong SAR China

**Keywords:** Forgotten Joint Score, Total knee arthroplasty, Patient report outcome, Early results, Ceiling effect, Floor effect

## Abstract

**Background:**

Forgotten Joint Score (FJS) has become a popular tool for total knee arthroplasty (TKA), but almost all studies had assessment performed 1 year after surgery. There is a need for a sensitive tool for earlier outcome assessment. The aim of this study was to investigate the usefulness of FJS within the first year after TKA.

**Methods:**

This was a cross-sectional study. Patients within the first year after primary TKA were recruited. FJS was translated into the local language with a cross-cultural adaptation and was validated by assessing the correlation with the Western Ontario and McMaster Universities Arthritis Index score (WOMAC). Ceiling and floor effects (highest or lowest 10% or 15%) of both scores were compared. Skewness of scores was assessed with a histogram.

**Results:**

One hundred sixty-three subjects were recruited: 84 (51.5%) had evaluation at 3 months after the operation, 56 (34.4%) at 6 months, and 23 (14.1%) at 12 months. FJS had fewer patients at the highest 10% (10.7% vs. 16.1%, *P* = 0.046) or 15% (19.6% vs. 32.1%, *P* = 0.027) at 6 months and within the first year overall (6.7% vs. 13.5%, *P* <0.001; 14.1% vs. 22.7%, *P* <0.001). Also, it had more patients at the lowest 10% (16.7% vs. 0%, *P* <0.001) or 15% (21.4% vs. 0%, *P* <0.001) at 3 months, 6 months (10.7% vs. 0%, *P* <0.001), and overall (12.9% vs. 0%, *P* <0.001; 16.6% vs. 0%, *P* <0.001). The skewness was much less than WOMAC (0.09 vs. −0.56).

**Conclusions:**

FJS has a low ceiling effect but a high floor effect in the first year after TKA. Such characteristics make it less useful for the general assessment of early patient report outcome after operation.

## Introduction

Forgotten Joint Score (FJS) has become a popular tool in assessing the outcome of total knee arthroplasty (TKA). It has been shown to have a good correlation with classic outcome scores like the Western Ontario and McMaster Universities Arthritis Index (WOMAC) score, the Knee Injury and Osteoarthritis Outcome Score (KOOS), and the Oxford Knee Score (OKS) [1–4]. A lower ceiling effect was observed in many studies [1–3, 5, 6], which suggests an advantage of this tool in assessing patients with good outcome. However, almost all studies on FJS were performed on patients more than 1 year after surgery [1, 7–10]. The rationale behind this was likely that the outcome of surgery had not reached plateau for assessment until 1 year after surgery. Nevertheless, Hiyama et al. [11] reported FJS improvement within 6 months after surgery reaching a plateau from 6 to 12 months. Also, with the increasing popularity of fast-track surgery, the need for early outcome assessment has become much greater. Newer surgical techniques, implant design, or perioperative protocol might result in subtle improvement in early outcome but with no long-term effect [12]. Despite this greater need for early and sensitive outcome assessment, appropriate tools for measurement are lacking [13] and evidence on the use of FJS earlier than 1 year is scarce [4, 5].

The aim of this study was to investigate the usefulness of FJS for assessing patients within the first year after TKA. The validity and reliability of FJS for early outcome assessment were investigated. In particular, the ceiling and floor effects of FJS were assessed. The null hypothesis of the study was that there is no difference in the ceiling and floor effects between FJS and WOMAC score at the early months after TKA.

## Materials and methods

### Subjects

This was a cross-sectional study. Patients who were within the first year after primary knee arthroplasty with post-operative evaluation in the out-patient clinic of our institute between July and September 2018 were recruited. Patients with dementia, psychiatric illness, and post-operative local complications like infection and fracture were excluded. All cases were performed with tourniquet during the whole procedure, medial parapatellar approach, posterior cruciate ligament sacrificed, cementation, and local infiltrative analgesia (30 mg ketorolac, 100 mg levobupivacaine, 0.5 mg adrenaline). Implants used were Attune (Depuy, Warsaw, IN, USA), Evolution (Microport, Arlington, TX, USA), Triathlon (Stryker, Mahwah, NJ, USA), or Legacy or Persona (Zimmer, Warsaw, IN, USA). Patients with four or more questions unanswered were also excluded from the whole study [3] and those with any question unanswered were excluded from testing for internal consistency. Institutional review board approval was obtained from the regional ethics committee (reference number: KW/EX-19-109(142–12)), and informed verbal consent was obtained from all patients.

### Outcome

All cases were evaluated by WOMAC score and FJS. Ceiling and floor effects of FJS and WOMAC score were assessed. They were defined as the proportion of patients scoring within the highest and the lowest percentile, respectively. Previous studies used either the 10th percentile [4] or the 15th percentile [3, 10]. The present study used both cutoff points to give a more detailed analysis. Validity of FJS was assessed by correlation with WOMAC score, which is a well-established outcome score widely used for total joint arthroplasty. Internal consistency within the questionnaire was assessed by correlation between all individual questions.

### Translation of FJS

Translation and cross-cultural adaptation were performed in the following steps: Two groups of translators were formed. Each group consisted of two bilingual researchers; one was an orthopedic surgeon and the other was an orthopedic nursing specialist. The English FJS was first translated independently by each member of the first group into two Cantonese Chinese versions (CC1 and CC2). The two versions were compared between the two translators and combined into a single Cantonese Chinese version (CC1–2).

Another group of bilingual researchers then “back-translated” the Cantonese Chinese version (CC1–2) into English again. Each of the translators would translate their individual version. The back-translated English versions were then compared with the original English questionnaire for “equivalence” in content, semantics, and concept by all of the involved translators. The process was repeated for those problematic questions whose meanings differ until equivalence was met. The adopted version after the above procedures would be administered to the first 20 subjects as a pilot version (CC-P) to assess its comprehensibility, fluency, and clarity. Owing to the high rate of illiteracy in elderly Cantonese, the questionnaire was to be read out word by word to illiterate patients by their relatives or helpers in the hospital. Since Cantonese Chinese is both a spoken and a written language, there would not be any discrepancy between comprehension by reading and hearing. The feedback from patients and helpers was used to make minor amendments to the final version (CC-F). This final version would then be subjected only to validity and reliability testing.

### Cross-cultural adaptation of FJS

In question 5, instead of asking the awareness of their artificial joint while travelling in a car, we have slightly modified the question to the awareness during any kind of traffic given the higher possibility of patients using public transport, which could be minivan, bus, train, and subway. In question 10, owing to the low popularity of gardening in our locality of Cantonese Chinese, the content about gardening was modified. Subjects were asked about awareness of their artificial joint while doing “trivial” activities instead of gardening. The idea was to measure the awareness while the subjects were engaged in activities that were not demanding physically (as in questions 11 and 12) but could distract them from their artificial joint.

### Validity and reliability of FJS

The convergent construct validity of the questionnaire was assessed by correlation of FJS with the WOMAC score, which is a well-established outcome score widely used for total joint arthroplasty. The degree of correlation was classified in accordance with Landis and Koch’s guidelines [14] of almost perfect (>0.8), substantial (0.6–0.8), moderate (0.4–0.6), fair (0.2–0.4), slight (0.0–0.2), or poor (0.0). Internal consistency was assessed by computerized calculation of correlation between all individual questions to give a correlation statistic called Cronbach’s α. It was classified as excellent (≥0.9), good (0.8–0.9), acceptable (0.7–0.8), questionable (0.6–0.7), poor (0.5–0.6), or unacceptable (<0.5).

### Statistical analysis

Correlation between FJS and WOMAC score was analyzed by Pearson test and reported as the Pearson correlation coefficient (R). Correlation between individual questionnaires was analyzed by Cronbach’s α. The difference in ceiling and floor effects was analyzed by chi-squared test. All tests were performed with IBM SPSS version 20.0 (IBM, Armonk, NY, USA). A *P* value of less than 0.05 was considered statistically significant.

## Results

### Validation

**Fig. 1:**
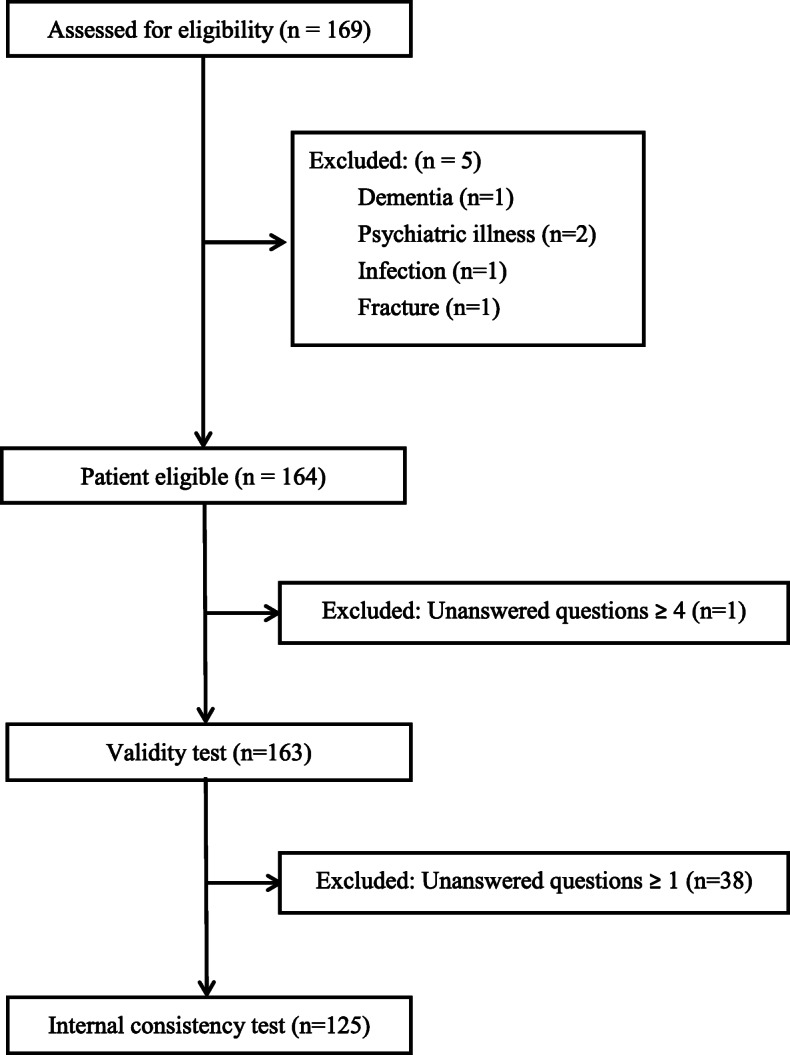
Flow diagram for patient selection

One hundred sixty-nine patients were evaluated after TKA during the study period. One case was excluded because of dementia, two because of psychiatric illness, one because of infection, and one because of fracture. Another case was excluded because of missing answers for four questions of FJS. In total, 163 subjects were recruited for validity testing (Fig. 1). Surgical implants used were Attune (24), Evolution (41), Triathlon (33), Legacy (45), and Persona (20). Thirty-eight cases did not complete all answers of FJS and were excluded for analysis for internal consistency. The mean age was 72 ± 7.5 (55–82) years, and 70.1% were female (Table 1). Eighty-four cases (51.5%) had evaluation at 3 months after the operation, 56 (34.4%) at 6 months, and 23 (14.1%) at 12 months. The overall response rate for all questions of FJS was 96.3% (88.4–100).

**Table 1:** Demographics and outcome scores

***n*** = 163	Mean	Range
Age, years	71.8 ± 7.5	51–88
Sex	Female 70.1%, male 29.9%	
Post-op, months	4.3 ± 3.8	1–12
WOMAC total	68 ± 18	23–96
WOMAC pain	14 ± 4	0–20
WOMAC stiffness	6 ± 2	1–8
WOMAC function	48 ± 12	15–68
FJS total	47 ± 29	0–100

*Abbreviations*: *FJS* Forgotten Joint Score, *WOMAC* Western Ontario and McMaster Universities Arthritis Index

Validity of the FJS was verified by moderate correlation between WOMAC score and FJS (R = −0.52, *P* <0.001) (Table 2). Excellent internal consistency was found for the 12 questions of FJS (Cronbach’s α: 0.96).

**Table 2:** Test validity and reliability of Forgotten Joint Score within 12 months after total knee arthroplasty

Dimension	Test	Subjects, n	Correlation	*P* value
Convergent construct validity	Pearson correlation	163		
WOMAC pain			−0.50	<0.001*
WOMAC stiffness			−0.42	<0.001*
WOMAC function			−0.51	<0.001*
WOMAC overall			−0.52	<0.001*
Internal consistency	Cronbach’s α	125	0.96	–

*Abbreviation*: *WOMAC* Western Ontario and McMaster Universities Arthritis Index

* *P* <0.05

### Ceiling, floor effects, and skewness

**Fig. 2:**
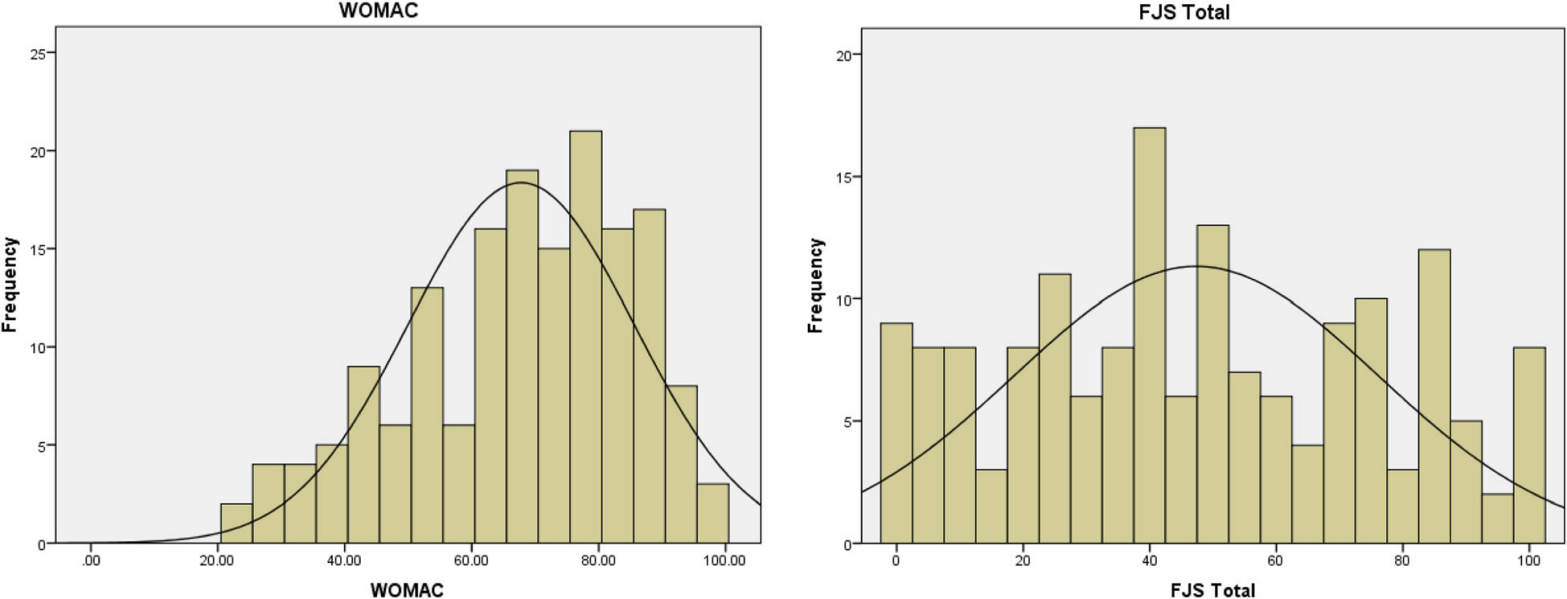
Histogram and skewness of FJS versus WOMAC. * *P* <0.05. *Abbreviations*: *FJS* Forgotten Joint Score, *WOMAC* Western Ontario and McMaster Universities Arthritis Index

FJS was found to have a lower ceiling effect than WOMAC score with a significantly lower proportion of patients among the highest 10% and 15% scores at 6 months and within the first year overall (Table 3). There was also a trend of lower percentage of ceiling in FJS at 12 months, although the difference was not statistically significant. On the other hand, a significantly higher floor effect at the first 6 months was found in FJS with a significantly higher proportion of patients scoring the lowest 10% and 15% at 3 months, 6 months, and within the first year overall. The skewness of FJS was significantly lower than WOMAC score (0.09 vs. −0.56) for the whole sample. The histogram of the whole sample showed obvious skewness to the left in WOMAC score and more even distribution in FJS (Fig. 2).

**Table 3:** Ceiling and floor effects within 12 months after total knee arthroplasty

	FJS, % (n)	WOMAC, % (n)	*P* value
**Ceiling**			
3 months (*n* = 84)			
Maximum	1.2 (1)	0 (0)	1
Highest 10%	2.4 (2)	7.1 (6)	1
Highest 15%	7.1 (6)	11.9 (10)	0.148
6 months (*n* = 56)			
Maximum	5.4 (3)	1.8 (1)	0.045*
Highest 10%	10.7 (6)	16.1 (9)	0.046*
Highest 15%	19.6 (11)	32.1 (18)	0.027*
12 months (*n* = 23)			
Maximum	8.7 (2)	8.7 (2)	0.170
Highest 10%	13.0 (3)	30.4 (7)	0.209
Highest 15%	26.1 (6)	39.1 (9)	0.162
Total (*n* = 163)			
Maximum	3.1 (5)	1.8 (3)	0.003*
Highest 10%	6.7 (11)	13.5 (22)	0.001*
Highest 15%	14.1 (23)	22.7 (37)	<0.001*
**Floor**			
3 months (*n* = 84)			
Minimum	6.0 (5)	0 (0)	0.061
Lowest 10%	16.7 (14)	0 (0)	<0.001*
Lowest 15%	21.4 (18)	0 (0)	<0.001*
6 months (*n* = 56)			
Minimum	5.4 (3)	0 (0)	0.248
Lowest 10%	10.7 (6)	0 (0)	0.030*
Lowest 15%	10.7 (6)	0 (0)	0.030*
12 months (*n* = 23)			
Minimum	0 (0)	0 (0)	1.000
Lowest 10%	4.3 (1)	0 (0)	1.000
Lowest 15%	13.0 (3)	0 (0)	0.248
Total (*n* = 163)			
Minimum	4.9 (8)	0 (0)	0.007*
Lowest 10%	12.9 (21)	0 (0)	<0.001*
Lowest 15%	16.6 (27)	0 (0)	<0.001*

*Abbreviations*: *FJS* Forgotten Joint Score, *WOMAC* Western Ontario and McMaster Universities Arthritis Index

* *P* <0.05

## Discussion

The most important finding from the present study is that FJS had a significantly lower ceiling effect and a higher floor effect within the first year after TKA. To the best of our knowledge, only two studies have investigated the use of FJS in the first year after TKA [4, 11]. In one of the studies, Hamilton et al. successfully validated FJS with OKS and noticed a lower ceiling effect [4], defined as scoring maximum 10% in FJS (12.6% vs. 25.5%). A more elaborate definition for ceiling or floor effect (that is, scoring the 10% and 15% highest or lowest score) was used by the present study. Also, this might be the first study to compare FJS with WOMAC score for the first 12 months after TKA. Consistent with the study by Hamilton et al., a lower ceiling effect of FJS was found even at 6 months after surgery. The finding was also consistent with the histogram in the present study, which showed a more even distribution of FJS, while WOMAC score was heavily skewed to the left. The skewness of FJS was much lower than WOMAC score (0.09 vs. −0.56). Other studies have shown a lower ceiling effect at longer post-operative periods. Behrend et al. [1] invented FJS and defined ceiling as scoring maximum in the new outcome scale. They first reported a lower percentage of ceiling in FJS (9.2%) compared with WOMAC score (16.7%–46.7%) in subjects 31 (15–58) months after TKA. Thompson et al. [2] also reported a lower ceiling effect (maximum score) in FJS than WOMAC score in subjects 39 months after TKA (6.8% vs. 9%). Similarly, Thomsen et al. [3] reported a lower ceiling effect in FJS than OKS (16% vs. 37%) in subjects 1–4 years after TKA. Ceiling was defined as scoring the highest 15% in their study. Using the same definition, Thienpont et al. [10] showed a lower ceiling effect of FJS compared with the quality-of-life section of the KOOS (9% vs. 29%). Another study by the same authors [8] showed a very large ceiling effect (maximum score) of 40%. However, more than half of the subjects in that study received partial knee arthroplasty, which could have affected the analysis to a large extent. In short, FJS seems to be a better tool to assess patients with good outcome in TKA even within the first year after surgery.

The floor effect of FJS was less well described in the literature. The present study might be the first to compare the floor effect of FJS with other outcome scores. Thienpont et al. [8] reported a floor effect of 16% of patients at least 1 year after TKA, but as mentioned above, the results of the study were confounded by partial knee arthroplasties. A study by Thienpont and Zorman showed a floor effect of 0–2% [9] at 1 year or more after TKA. However, the results were not compared with other scoring systems. Cao et al. [6] reported no floor effect of FJS in subjects at an average of 28  months (12–94) after TKA. But there was no comparison with other scoring systems. The present study demonstrated a significantly higher floor effect in FJS than WOMAC score in the first 6 months after TKA. This was understandable given the natural course of recovery after TKA, which usually takes 3–6 months for early symptoms due to surgery to resolve. Hiyama et al. [11] attributed this to the presence of pain in the first month. In this regard, FJS seems not to be a good choice to assess patients with worse outcome, particularly in the first 6 months after surgery.

Other findings of the present study included excellent response rate and internal consistency of FJS within 1 year from TKA. These were consistent with other studies on the use of FJS after 1 year from TKA. Also, the validity of the tool was confirmed by significant correlation with WOMAC score. The moderate correlation of 0.52 was not as high as reported in other studies (0.7–0.79) [1, 2]. This could be explained by the large difference in ceiling and floor effect between two scoring systems noticed in the first year in the present study.

Overall, the present study suggests that FJS is a useful tool in assessing the superiority of early results after knee arthroplasty. It might be particularly useful where differences in good outcome are not easily detectable because of subtle differences in prosthesis design, surgical technique, or post-operative regime. It might also be particularly valuable when the differences only lie in the early phase after surgery, such as comparison of the outcome of different practices of fast-track surgery. However, owing to the higher floor effect in the first 6 months after surgery noticed in FJS in the present study, one should be mindful of such limitations of this tool. It is likely not to be a proper tool for early detection or comparison of poor outcome in TKA. A better tool may be required for an overall assessment of early patient report outcome.

There were some limitations of the present study. First, the number of patients having scores at 12 months was small. This might have rendered the absence of significant results at 12 months. Second, owing to the expected increase in FJS during the first 6 months, test-retest reliability was not assessed in the present study [11]. Nevertheless, it has been found to be excellent by other studies [1–3]. Also, body mass index, preoperative alignment, and symptom duration might affect the final FJS but they were not analyzed in the present study. This might have affected the ceiling or floor effect should they have deviated grossly from normal distribution in the recruited sample. Another limitation of the present study was that the ceiling and floor effect of individual questions within the FJS was not assessed. The knowledge of this might help the development of a better measurement tool to assess early outcome of knee arthroplasty surgery.

## Conclusion

FJS has a low ceiling effect but a very high floor effect in the first year after TKA. Such characteristics make it a useful tool specifically in comparing the superiority of early results after knee arthroplasty but it is less useful for general assessment of early patient report outcome after an operation.

## Data Availability

The data that support the findings of this study are available, but consent to share them with the public or a third party has not been obtained.

## Abbreviations

FJS: Forgotten Joint Score
KOOS: Knee Injury and Osteoarthritis Outcome Score
OKS: Oxford Knee Score
TKA: Total knee arthroplasty
WOMAC: Western Ontario and McMaster Universities Arthritis Index
